# Evaluation of a school-based, experiential-learning smoking prevention program in promoting attitude change in adolescents

**DOI:** 10.18332/tid/134605

**Published:** 2021-06-18

**Authors:** Dimitra P. Mpousiou, Nikolaos Sakkas, Elpidoforos S. Soteriades, Michalis Toumbis, Stavros Patrinos, Anna Karakatsani, Areti Karathanassi, Vasilios Raftopoulos, Christina G. Gratziou, Paraskevi A. Katsaounou

**Affiliations:** 1 National and Kapodistrian University of Athens, Athens, Greece; 2 Department of Physiotherapy, Faculty of Life Sciences and Medicine, King’s College London, London, United Kingdom; 3 Healthcare Management Program, Faculty of Economics and Management, Open University of Cyprus, Nicosia, Cyprus; 4 Institute of Public Health, College of Medicine and Health Sciences, United Arab Emirates University, Al Ain, United Arab Emirates; 5 Environmental and Occupational Medicine and Epidemiology, Department of Environmental Health, Harvard T.H. Chan School of Public Health, Harvard University, Boston, United States; 6 School of Medicine, European University Cyprus, Nicosia, Cyprus; 7 Hellenic National Public Health Organization, Athens, Greece; 8 2nd Pulmonary Clinic, Atticon University General Hospital, Athens, Greece; 9 Educational Institute, Athens, Greece; 10 Smoking Cessation Centre, Evgenidio Hospital, Athens, Greece; 11 School of Medicine, National and Kapodistrian University of Athens, Athens, Greece; 12 Evaggelismos Hospital, National and Kapodistrian University of Athens, Athens Greece

**Keywords:** smoking, prevention, adolescents, experiential learning, school

## Abstract

**INTRODUCTION:**

School-based tobacco control programs exhibit great variety. Our study aimed to evaluate the effectiveness of an experiential learning smoking prevention program in facilitating knowledge acquisition, forging healthy attitudes, and decreasing intention to smoke.

**METHODS:**

A school-based intervention-control study was implemented during the 2016–2017 academic year among middle-school students in Athens, Greece. The experiential learning intervention was delivered using an interdisciplinary approach, bridging excerpts from ancient classical Greek myths, Aesop fables and ancient classical literature (Aristotle, Herodotus, Plutarch, Xenophon, Homer’s Epics), with their decoded archetypal symbols applied in a smoking and tobacco control paradigm. An anonymous self-administered questionnaire was used at baseline and at follow-up at 3 months to evaluate program effectiveness.

**RESULTS:**

A total of 351 students participated in our study; 181 (51.6%) in the intervention group and 170 (48.4%) in the control group. The mean age of student participants was 13 years (SD=0.96). Students in the intervention group were more likely to improve their knowledge of the adverse effects of smoking, develop attitudes against smoking and report a negative intention to smoke in the first year following the intervention, compared to the control group.

**CONCLUSIONS:**

This study provides evidence that school-based experiential learning smoking prevention programs improve smoking-related knowledge, enhance anti-smoking attitudes and reinforce negative intentions toward tobacco products.

## INTRODUCTION

Smoking remains a primary cause of morbidity and mortality and constitutes one of the most significant public health challenges of our time^1^. It can lead to a myriad of adverse health consequences, including cardiovascular disease, chronic respiratory health problems, and many cancers, to mention a few. The use of cigarettes and other tobacco products account for more than 6 million deaths annually worldwide, with smoking affecting developed and developing populations alike^2^.

One of the most alarming findings concerning the occurrence of smoking initiation in early adolescence is that more than 90% of adult smokers begin smoking at an age younger than 18 years^3^. Early experimentation has been associated with a higher risk of nicotine dependence, leading to nicotine addiction that extends into adulthood^4^. Given the above observations, the implementation of tobacco use prevention programs in schools are of primary importance and require overall prioritization. Efforts should focus on early intervention, with tobacco control initiatives targeting the younger generation^5^.

Preventative programs in schools display broad heterogeneity, focusing on traditional informational booklet distribution and delivery of class-based educational sessions^6^ to contemporary approaches that focus on the cultivation of personal skills that challenge negative smoking influences^7^.

Examples of such programs include the Social Psychological Deterrents of Smoking in Schools Project^8^, The North Karelia Youth Project^9^, the Minnesota Smoking Prevention Program^10^, the Project SHOUT (Students Helping Others Understand Tobacco)^11^, the Tobacco and Alcohol Prevention Program (TAPP)^12^, the Life Skills Training (LST) project^13^, the Project Towards No Tobacco Use (Project TNT)^14^, the Stay Away from Tobacco (SAFT) program^15^, and the Drug Abuse Resistance Education (DARE) program^16^.

While the content of preventative programs is important, delivery and implementation methods retain equal significance, influencing the effectiveness of interventions. Experiential learning is characterized by a student-centered educational approach that enables active student participation in teacher-led, transformational activities^17^. In essence, it utilizes previously acquired experiences, such as daily encounters and real-life scenarios, to influence personal attitude change. Strategies implemented in class may pivot around active dialogue, role-playing, productive debate, and peer-led teaching. Experiential learning has been shown to improve participation, enhance educational experiences and promote long-lasting enlightenment^18,19^. Unfortunately, few experiential learning programs have been previously reported in general health or smoking prevention literature, despite promising efficacy.

The proposed and widely used framework of experiential learning, called 4-H, reflects an active educational approach. This approach facilitates the participation and collaboration of students and adults and comprises skill-learning exercises and peer-led sessions^20^. One of the few experiential learning programs reported in wider literature was by Onion and Bartzokas^21^ who employed an interactive educational strategy to increase participation. Results demonstrated an enduring attitude change among primary care physicians in complying with evidence-based guidelines for optimal infection treatment^22^. Experiential learning programs on school-centered tobacco control are less frequently reported. Norman et al.^23^ tried the value of a theory-based experiential intervention in encouraging smoking cessation. They found that a smoking intervention was more effective than control in prompting immediate motivation to quit^22,23^.

Previous, successful tobacco control programs were considered when creating this individualized, experiential learning program for middle- and high-school students. We designed a multi-faceted program, incorporating characteristics from traditional programs, such as education about the harmful effects of smoking and newer programs that focus on developing associated personal and social skills. We adopted an interactive, experiential-learning process, assuming different elements from ancient Greek literature and mythology.

Our study aimed to implement and evaluate the effectiveness of a smoking prevention program in facilitating knowledge acquisition, forging healthy attitudes against smoking and decreasing intention to smoke among middle- and high-school students in Athens, Greece.

## METHODS

The study was conducted during the 2016–2017 academic year among middle-school students in Athens, Greece. Permission to implement the study was obtained from the Greek Ministry of Education, and ethical approval was granted by the Bioethics Committee of Evangelismos Hospital. The study was supervised by faculty members of the first ICU clinic of Evangelismos Hospital, National and Kapodistrian University of Athens (16/6/2016, Protocol Number 131).

### Study participants

The study was fulfilled in five selected middle schools. Students from 1st to 3rd grade in middle-school were invited to participate in intervention and control groups. The final study group was a convenient sample obtained from participating middle schools. Among 407 students from different classes that were randomly allocated to intervention (206) or the control group (201), 56 were excluded since there was no parental approval, were absent once, or completed incorrectly the questionnaires. Finally, 170 students were enrolled in the control group and 181 in the intervention group.

The experiential tobacco control intervention took place in school classrooms of 20–25 students during their everyday program and lasted for two school hours plus the in-between break. Students from both groups completed the questionnaires twice. Once before and secondly 3 months after the intervention. Data collection lasted about seven months (the academic year 2016–2017) at five public schools of the northwestern suburbs of Athens after the protocol was presented and approved by each school director.

### Interventions

The Theory of Reasoned Action and Planned Behavior, accepted as a fundamental psychological concept in the prediction of health-related behavior, formed the overarching framework of our study.

We wished to design and implement an innovative and attractive experiential learning prevention program for adolescent students, incorporating new elements that could meet their developmental needs without provoking resistance from their part. We designed and configured our intervention to give the best result in three axes: the provision of knowledge, the recognition of social influences and the development of personal resistance skills, including not accepting cigarettes from peers. The first axis consisted of a presentation on respiratory health, nicotine dependence and the immediate adverse effects of smoking. The other two experiential learning axes were achieved interactively through an interdisciplinary approach. Our educational material consisted of excerpts from the large repository of the ancient and classical Greek literature (Homeric epics, works by Aristotle, Herodotus, Plutarch), the Aesop Fables as well as selections from modern Greek poetry, where archetypal symbols were decoded and applied in tobacco control paradigms. We utilized their didactic character, their reference to human values, their symbolic content, and their heroes’ vivid figures in our intervention, including role-playing to build personal-centered social skills (resistance to peer pressure, building self-efficacy etc.), and instructor-led, pre-designed, student debates. For example, the Aesop Fable ‘Fox Without Tail’ was used in order to develop skills of resistance to peer pressure and social stereotypes, the ancient Greek myth ‘Hercules at the Crossroads: Vice and Virtue’ aimed at developing proper judgement, the adventures of Odysseus on the island of Aeolus and the island of the Sirens, pointed to the irreversible nature of choices such as the concept of addiction. Cavafy’s poems ‘The windows’ and ‘Walls’ that negotiate personal and inner freedom, free will and free choice were used in order to highlight the loss of autonomy that occurs with the onset of smoking.

### Data collection

Change in knowledge, attitude, and intention to smoke, was evaluated before and after the intervention. A pre-post test questionnaire was administered at baseline and at follow-up at 3 months, respectively. Data were collected through an anonymous self-administered questionnaire, distributed to all students in both groups. The questionnaire covered demographic information and student smoking behavior, in addition to knowledge, attitudes, and intention to smoke. The final questionnaire integrated items from the Global Youth Tobacco Survey (GYTS - Standard Core Questionnaire)^24^, the Theory of Reasoned Action and Planned Behavior questionnaire^25^, and the questionnaire from another study^26^.

### Statistical analysis

Data were entered into a computerized database and analyzed using the Statistical Package for Social Sciences (IBM SPSS V.26). Frequencies and percentages, and means and standard deviations were calculated for descriptive purposes. A chi-squared test was used for categorical variables, while a t-test and Analysis of Variance (ANOVA) were used for continuous variables. To discretely assess students’ knowledge, attitudes and intention toward smoking, a total score was calculated for each outcome. For corresponding questions: positive answers were tabulated as 1, negative answers as -1, and neutral answers as 0. The score of the knowledge and attitudes scale takes values from -16 (incomplete knowledge and attitudes) to +16 (full knowledge and attitudes). Attitudes scores ranged from -9 to +9, respectively. Regression analysis was employed to examine the intervention effect size of knowledge and attitudes, as well as the impact of other factors such as age, gender, and baseline scores on pre- and post-intervention scores. Specifically, the difference of the total score of pre- and post-measurement on knowledge (first model) and attitudes (second model) was the dependent variable of each linear regression analysis, respectively. Independent variables were the students’ age depicted by the grade, gender, group (intervention or control) and the pre-measurement total score of knowledge and attitudes, respectively for the above models. A two-tailed p-value of ≤0.05 was considered statistically significant.

## RESULTS

A total of 351 students participated in our study, with 181 (51.6%) in the intervention group and 170 (48.4%) in the control group. The mean age of the participants was 13.0 years (SD=0.96) years; 12.8 years (SD=0.9) in the intervention and 13.3 years (SD=0.95) in the control group (p<0.001). The majority of the participants were females (191; 54.5%), and the distribution of middle-school students across grades was 202 (57.5%) in Grade 1, 68 (19.4%) in Grade 2, and 81 (23.1%) in Grade 3. There was a significant difference in age distribution between the intervention and control groups (p<0.001) depicted by the school grade. Two out of three (69%) of first grade students were in the intervention group versus 45.3% in the control group. There were more students in second and third grade in the control group (55.7%) versus 31% of the intervention group. Non-smoking adolescents were at the same level in both groups (81.8%, p=0.91). These results are described in more details in Table 1. Regarding family smoking, there was no statistically significant difference between intervention and control group in all categories depicted in Table 1. For none, mother, father, both, other member and ex-smoking in the intervention group the results were (50.6, 54.0, 53.8, 53.8, 56.1, 39.5 and 44.8%) and in the control group they were (49.4, 46.0, 46.2, 43.8, 60.5 and 55.2%). Regarding friends’ smoking rates, there was no statistically significant difference between the intervention and the control group (p=0.150).

**Table 1:** Demographic and other characteristics of 351 students

*Characteristics*	*Total (N=351) n (%)*	*Intervention (n=170) n (%)*	*Control (n=181) n (%)*	*p*
**Mean age**, mean ± SD	13.0±0.96	12.76±0.91	13.26±0.95	<0.001
**Gender**				
Male	160	73 (42.9)	87 (48.1)	0.33
Female	191	97 (57.1)	94 (51.9)	
**Grade**				
1st	202	125 (69.0)	77 (45.3)	
2nd	68	30 (16.6)	38 (22.4)	<0.001
3rd	81	26 (14.4)	55 (32.3)	
**Smoking status**				
No	287	148 (81.8)	139 (81.8)	
Tried	49	26 (14.3)	23 (13.5)	0.91
More than once	15	7 (3.9)	8 (4.7)	
**Family smoking**				
None	172	87 (50.58)	85 (49.42)	0.717
Mother	113	61 (53.98)	52 (46.02)	0.552
Father	119	64 (53.78)	55 (46.22)	0.533
Both	57	32 (56.14)	25 (43.86)	0.450
Other member	38	15 (39.47)	23 (60.53)	0.114
Ex-smoking	67	30 (44.77)	37 (55.23)	0.216
**Friends’ smoking**				
None	273	140 (77.3)	133 (78.2)	
Best friend	6	5 (2.8)	1 (0.6)	
Some	68	36 (19.9)	32 (18.8)	0.150
Most	1	0 (0)	1 (0.6)	
All	3	0 (0)	3 (1.8)	

A statistically significant improvement in knowledge acquisition in the intervention group was observed (prescore 5.6±2.1, postscore 7.7±1.7, p<0.001). In contrast, there was a statistically significant decrease in knowledge in the control group (prescore 6.5±1.7, postscore 6.1±1.8, p=0.005). Gender analysis revealed the above statistically significant improvement in knowledge in the intervention group. In boys, the prescore was 5.6±2.2, whilst the postscore was 7.7±1.3 (p<0.001). In girls, similar results were found (Table 2).

**Table 2:** Outcome measures pre- and post-intervention in both student groups (N=351)

*Outcomes*	*Intervention (n=170)*			*Control (n=181)*		
	*pre*	*post*	*p*	*pre*	*post*	*p*
**Knowledge on adverse effects of smoking**						
Total score	5.6±2.1	7.7±1.7	<0.001	6.5±1.7	6.1±1.8	0.005
Boys	5.6±2.2	7.7±1.3	<0.001	6.6±1.8	6.2±1.9	0.040
Girls	5.6±2.6	7.7±1.1	<0.001	6.5±1.8	6.2±1.8	0.123
1st Grade	5.8±2.1	7.6±1.3	<0.001	6.4±1.7	6.6±1.9	0.292
2nd Grade	5.0±2.0	8.0±0.8	<0.001	6.5±1.8	5.8±1.7	<0.001
3rd Grade	5.2±2.2	7.8±1.1	<0.001	6.7±1.1	6.6±1.8	0.597
**Attitudes against smoking**						
Total score	5.3±2.7	7.1±2.1	<0.001	6.0±2.5	5.5±3.0	0.014
Boys	4.9±2.9	7.1±1.9	<0.001	5.2±2.4	4.5±3.2	0.019
Girls	5.7±2.6	7.1±2.4	<0.001	6.6±2.4	6.3±2.7	0.264
1st Grade	5.5±2.7	7.3±2.3	<0.001	6.4±2.3	5.6±3.2	<0.001
2nd Grade	4.9±3.1	6.7±2.0	<0.001	5.7±2.9	5.9±2.9	0.521
3rd Grade	4.6±2.4	6.8±1.7	<0.001	5.7±2.5	5.3±3.1	0.177
Intention to smoke in the next 12 months*						
Unlikely	147 (81.2)	171 (94.4)		140 (82.4)	128 (75.3)	
Likely	30 (16.6)	9 (5.0)	*	23 (13.5)	35 (20.6)	*
Very likely	4 (2.2)	1 (0.6)		7 (4.1)	7 (4.1)	

Numbers are given as either mean ± standard deviation or n (%).

* There was a statistically significant difference between intervention and control group with respect to post intervention intention to smoke (p<0.001).

In all grades, a statistically significant improvement in knowledge level was found in the intervention group (p<0.001). The highest increase depicted in the 2nd grade, in which the prescore was 5±2 and the postscore was 8±0.8. In this grade was found a slight decrease in postscore (5.8±1.7) compared to prescore (6.5±1.8) in the control group, which was statistically significant (p<0.001). Similar results were found at pre- and post-intervention scores (Table 2), in grades 1st and 3rd, whilst in the control group in 1st grade, the prescore was 6.4±1.7 and the postscore was 6.6±1.9 (p=0.292). In the 3rd grade, the prescore was 6.7±1.1 and the postscore was 6.6±1.8 (p=0.597).

The attitudes total postscore was higher in the intervention group (7.1±2.1) compared to the prescore (5.3±2.7) (p<0.001). In the control group, there was a statistically significant decrease in total score (p=0.014). Boys prescore was 4.9±2.9 and postscore was 7.1±1.9 in the intervention group (p<0.001). Girls prescore was 5.7±2.6 and postscore was 7.1±2.4 in the intervention group (p<0.001). It is valuable to refer to the boys’ attitudes were at lower level in prescore compared to girls, but the postscore was at the same level. Boys achieved lower postscore in the control group at a statistically significant level (prescore 5.2±2.4 vs postscore 4.5±3.2, p=0.019). Girls achieved a lower postscore (6.3±2.7) in the control group (vs prescore 6.6±2.4) but not at a statistically significant level (p=0.264). In the 1st grade, the attitudes prescore and postscore were higher than in other grades. In all grades, pre and post attitudes scores were statistically significant (p<0.001) (Table 2). In 1st grade pre and post attitudes scores were 5.5±2.7 and 7.3±2.3, in 2nd grade they were 4.9±3.1 and 6.7±2.0, and in 3rd grade they were 4.6±2.4 and 6.8±1.7, in the intervention group, respectively. The scores of the control group were 6.4±2.3 and 5.6±3.2 for 1st grade (p<0.001), for 2nd grade they were 5.7±2.9 and 5.9±2.9 (p=0.521) and for 3rd grade they were 5.7±2.5 and 5.3±3.1 (p=0.177), respectively.

Obviously, taking into account the above results, we observed a decrease in intention to smoke in the intervention group. A significant increase in the percentage of students reporting that they were unlikely to smoke in the year following in the intervention group (94.4% vs 81.2%, p<0.001) was observed. It is remarkable to report the statistically significant (p<0.001) decrease of the negative intension to smoke in the future at the control group (82.4% vs 75.3%).

In Table 3, we delineate the results of the linear regression model, examining the effect of the intervention on knowledge and attitudes scores. These were adjusted according to baseline scores, school grade (as a proxy for age), and gender. All independent variables remained in both models due to the medical impact and despite the obvious no statistically significant impact of gender on knowledge score and the grade additionally to the gender on the attitudes score. We found that the intervention also had a significant effect on knowledge acquisition and attitude change in the adjusted, multi-variable regression model. The knowledge difference score was higher in grade 3 students compared to grade 1 students by 0.4722 units (p=0.012). In the control group the knowledge difference score was lower compared to the intervention group by 1.9681 units (p<0.001). For each unit of increase in pre knowledge score, the difference decreases by 0.6392 units (p<0.001). These results are referred at the baseline of 5.6068 units of knowledge difference score, taking into account all included parameters as independent variables. Similarly, the attitudes difference score was lower in the control group compared to the intervention group by 1.9212 units (p<0.001). For each unit of increase in pre attitudes score, the difference decreases by 0.5086 units (p<0.001). These results are referred at the baseline of 4.3335 units of attitudes difference score taking into account all the model parameters.

**Table 3:** Predictors of the difference between total post- and pre- intervention scores in knowledge and attitudes of 351 students in Athens

	*Beta coefficient*	*S.E.*	*t*	*p>t*	*95% CI*	
**Knowledge difference score**						
Grade	0.2242	0.0916	2.45	0.015	0.044	0.404
Gender	-0.0014	0.1478	-0.01	0.993	-0.292	0.289
Intervention	-1.9712	0.1563	-12.61	<0.001	-2.279	-1.664
Baseline score	-0.6377	0.0376	-16.96	<0.001	-0.712	-0.564
Constant	7.3348	0.3157	23.24	<0.001	6.714	7.956
**Knowledge difference score (Explanation model)**						
Grade 2	0.1119	0.1957	0.57	0.568	-0.273	0.497
Grade 3	0.4722	0.1870	2.53	0.012	0.104	0.840
Female	0.0076	0.1485	0.05	0.959	-0.285	0.300
Control group	-1.9681	0.1565	-12.57	<0.001	-2.276	-1.660
Baseline score	-0.6392	0.0377	-16.95	<0.001	-0.713	-0.565
Constant	5.6068	0.2546	22.02	<0.001	5.106	6.108
**Attitudes difference score**						
Grade	-0.0148	0.1540	-0.10	0.924	-0.318	0.288
Gender	0.3165	0.2520	1.26	0.210	-0.179	0.812
Intervention	-1.9170	0.2568	-7.46	<0.001	-2.422	-1.412
Baseline score	-0.5105	0.0478	-10.69	<0.001	-0.604	-0.417
Constant	6.2908	0.4831	13.02	<0.001	5.341	7.241
**Attitudes difference score (Explanation model)**						
Grade 2	0.1623	0.3281	0.49	0.621	-0.483	0.808
Grade 3	-0.0658	0.3138	-0.21	0.834	-0.683	0.552
Female	0.3003	0.2536	1.18	0.237	-0.199	0.799
Control group	-1.9212	0.2571	-7.47	<0.001	-2.427	-1.415
Baseline score	-0.5086	0.0479	-10.62	<0.001	-0.603	-0.414
Constant	4.3335	0.3287	13.18	<0.001	3.687	4.980

## DISCUSSION

Our intervention was experienced and mastered by students who shared it with their classmates, understanding and describing its meaning. They identified, discussed and analyzed the key points through reflection, decoding of the symbols and their projection in similar situations. Then they generalized and related the experience and skills they acquired with everyday life, connecting them with examples from real life. Finally, they applied what they learned in similar situations^20^.

Our intervention was effective among adolescents aged 12–15 years, a vulnerable and critical period for tobacco experimentation and uptake. Students in the intervention group were more likely to improve their awareness of the effects of smoking, adopt attitudes against smoking and less likely to smoke in the first year following the intervention.

Increased awareness has been associated with a decrease in motivation to smoke and a lower probability of tobacco uptake. In contrast, a lack of awareness may lead to complacency and carelessness for unhealthy smoking behavior^27^. Our study findings resulted in significantly enhanced knowledge of the direct, imminent, and tangible consequences of smoking and are in line with other international reports demonstrating that school-based programs are efficient in knowledge acquisition^28^. This program aimed at understanding both the adverse effects of intermittent tobacco use and passive (secondhand and thirdhand) smoking. Intermittent tobacco use can lead to the development of regular smoking in adulthood^29^, while comprehension of the dangers of passive smoking can protect teenagers from unnecessary exposure, prevent habitual tobacco use and encourage individuals to respect no-smoking rules at home^3^.

Negative attitudes towards tobacco significantly improved at the follow-up at 3 months among the intervention group. Improvement was observed in questions relating to attitudes about the perceived benefits of and social stigma surrounding smoking among teenagers (e.g. acceptance and recognition from peers). A positive attitude toward tobacco use has been shown to correspond to a high intention to smoke^30^, an increased frequency of tobacco uptake, and a continuation of smoking into adulthood^31^. In line with relevant studies, our experiential learning program was effective in increasing the percentage of participants who reported intention to smoke as ‘unlikely’ at follow-up. There was also a significant reduction in students reporting intention to smoke as ‘very likely’, which may translate to a decreased chance of actual future tobacco uptake, according to the Theory of Planned Behavior^32^.

### Limitations

Limitations of the present study include the use of a subjective, self-reported questionnaire, and a lack of cross-examination using an objective laboratory biomarker, such as cotinine. Notwithstanding, low metabolite levels, coupled with an irregular pattern of tobacco use among adolescents, challenge the validity of biochemical markers. In this context, self-reported tools are considered an equally reliable method of analyzing smoking behavior^33^. To avoid performance bias, the experiential learning intervention was carried out by the same investigator in all class sessions. Further, the effectiveness of our intervention was recorded at follow-up at 3 months, which could raise questions about its long-term efficacy. School-based studies with a prolonged follow-up are generally difficult and impractical, given limitations of the academic calendar. Nevertheless, existing literature suggests that effective short-term, school-based programs may have potential lasting effects^34^.

Additionally, although the student participants were enrolled from a ‘convenient sample’, we believe that the selected student population is relatively representative of the students attending public schools in Athens because the student population of the public education system is more or less homogeneous with respect to the socioeconomic status and school performance.

Data collection was conducted only for students present on the day of the intervention and at follow-up. Per wider literature, absent students tend to demonstrate lower academic achievement and appear more susceptible to addictive behaviors, potentially skewing study results^35^. In the present study there was no significant difference in absence between the two groups. Finally, the mean age of students in the intervention group was slightly lower than that of the control group, which may have influenced the prevalence of smoking and students’ receptiveness to anti-smoking messages. That said, the proportion of student smokers in our study was similar in both groups. Additionally, we found that the effect of the intervention was greater in the 2nd grade which was represented almost equally in both groups.

## CONCLUSIONS

The findings of our study support our initial hypothesis; school-based, experiential learning smoking prevention programs are effective in augmenting smoking-related knowledge, enhancing anti-smoking attitudes, and minimizing intention to initiate or continue smoking. All outcome measures demonstrated a significant increase in the intervention group at three months post-implementation. Group differences can be attributed to the intervention itself and agree with similar studies, most of which support the efficacy and superiority of newer experiential learning programs. The current study is the first to utilize an interdisciplinary approach and use ancient Greek literature, myths and poems as educational tools.

Future research is needed to explore the effects of such a program in larger adolescent samples over an extended follow-up period, and further, explore its utility through parental involvement and the use of mass media. Besides, appraisal of the current program in settings outside formal education may expand its scope. Consideration of the above parameters will reinforce the current findings, augment our understanding of adolescent smoking patterns and encourage the design of future school-based interventions. Experiential smoking prevention programs may constitute a successful strategy in preventing adolescent tobacco uptake and form a cornerstone of public health policies against the tobacco epidemic.

## References

[1] Murray CJ, Lopez AD (1997). Global mortality, disability, and the contribution of risk factors: Global Burden of Disease Study. Lancet.

[2] The World Bank (1999). Curbing the epidemic: governments and the economics of tobacco control. Tob Control.

[3] (1994). Guidelines for school health programs to prevent tobacco use and addiction. J Sch Health.

[4] Taioli E, Wynder EL (1991). Effect of the age at which smoking begins on frequency of smoking in adulthood. N Engl J Med.

[5] Vickers KS, Thomas JL, Patten CA, Mrazek DA (2002). Prevention of tobacco use in adolescents: review of current findings and implications for healthcare providers. Curr Opin Pediatr.

[6] Thomas RE, McLellan J, Perera R (2015). Effectiveness of school-based smoking prevention curricula: systematic review and meta-analysis. BMJ Open.

[7] Flay BR (1985). Psychosocial approaches to smoking prevention: a review of findings. Health Psychol.

[8] Evans RI, Rozelle RM, Mittelmark MB, Hansen WB, Bane AL, Havis J (1978). Deterring the Onset of Smoking in Children: Knowledge of Immediate Physiological Effects and Coping with Peer Pressure, Media Pressure, and Parent Modeling. J Appl Soc Psychol.

[9] Vartiainen E, Fallonen U, McAlister AL, Puska P (1990). Eight-year follow-up results of an adolescent smoking prevention program: the North Karelia Youth Project. Am J Public Health.

[10] Arkin RM, Roemhild HF, Johnson CA, Luepker RV, Murray DM (1981). The Minnesota smoking prevention program: a seventh-grade health curriculum supplement. J Sch Health.

[11] Elder JP, Wildey M, de Moor C (1993). The long-term prevention of tobacco use among junior high school students: classroom and telephone interventions. Am J Public Health.

[12] Hansen WB, Malotte CK, Fielding JE (1988). Evaluation of a tobacco and alcohol abuse prevention curriculum for adolescents. Health Educ Q.

[13] Botvin GJ, Eng A (1982). The efficacy of a multicomponent approach to the prevention of cigarette smoking. Prev Med.

[14] Sussman S, Dent CW, Stacy AW, Hodgson CS, Burton D, Flay BR (1993). Project Towards No Tobacco Use: implementation, process and post-test knowledge evaluation. Health Educ Res.

[15] Chen X, Fang X, Li X, Stanton B, Lin D (2006). Stay away from tobacco: a pilot trial of a school-based adolescent smoking prevention program in Beijing, China. Nicotine Tob Res.

[16] Ahmed NU, Ahmed NS, Bennett CR, Hinds JE (2002). Impact of a Drug Abuse Resistance Education (D.A.R.E) Program in Preventing the Initiation of Cigarette Smoking in Fifth- and Sixth-Grade Students. J Natl Med Assoc.

[17] Bonwell CC, Eison JA (1991). Active Learning: Creating Excitement in the Classroom. ASHE-ERIC Higher Education Reports.

[18] Bonwell CC, Sutherland TE (1996). The active learning continuum: Choosing activities to engage students in the classroom. New Directions for Teaching and Learning.

[19] Chiu PHP, Cheng SH (2016). Effects of active learning classrooms on student learning: a two-year empirical investigation on student perceptions and academic performance. Higher Education Research and Development.

[20] Blyler K (2014). The Power of Experiential Learning. Memoirs of Study in Aquatic and Marine Environmental Education.

[21] Onion CW, Bartzokas CA (1998). Changing attitudes to infection management in primary care: a controlled trial of active versus passive guideline implementation strategies. Fam Pract.

[22] Simmons VN, Heckman BW, Fink AC, Small BJ, Brandon TH (2013). Efficacy of an experiential, dissonance-based smoking intervention for college students delivered via the internet. J Consult Clin Psychol.

[23] Simmons VN, Brandon TH (2007). Secondary smoking prevention in a university setting: a randomized comparison of an experiential, theory-based intervention and a standard didactic intervention for increasing cessation motivation. Health Psychol.

[24] Global Youth Tobacco Survey Collaborative Group (2014). Global Youth Tobacco Survey (GYTS): Core Questionnaire with Optional Questions, Version 1.2.

[25] Ajzen I (2019). Constructing A Theory of Planned Behavior Questionnaire.

[26] Charlton A (1984). Children's opinions about smoking. J R Coll Gen Pract.

[27] Song AV, Morrell HE, Cornell JL (2009). Perceptions of smoking-related risks and benefits as predictors of adolescent smoking initiation. Am J Public Health.

[28] Bruvold WH (1990). A meta-analysis of the California school-based risk reduction program. J Drug Educ.

[29] Fidler JA, Wardle J, Brodersen NH, Jarvis MJ, West R (2006). Vulnerability to smoking after trying a single cigarette can lie dormant for three years or more. Tob Control.

[30] Meier KS (1991). Tobacco truths: the impact of role models on children's attitudes toward smoking. Health Educ Q.

[31] Najem GR, Batuman F, Smith AM, Feuerman M (1997). Patterns of smoking among inner-city teenagers: smoking has a pediatric age of onset. J Adolesc Health.

[32] Ajzen I, Fishbein M (2002). Attitudes and The Attitude-Behavior Relation: Reasoned and Automatic Processes. Eur Rev Soc Psychol.

[33] Dolcini MM, Adler NE, Lee P, Bauman KE (2003). An assessment of the validity of adolescent self-reported smoking using three biological indicators. Nicotine Tob Res.

[34] Flay BR (2009). The promise of long-term effectiveness of school-based smoking prevention programs: a critical review of reviews. Tob Induc Dis.

[35] Reimers TM, Pomrehn PR, Becker SL, Lauer RM (1990). Risk factors for adolescent cigarette smoking. The Muscatine study. Am J Dis Child.

